# Effect of Cancer Treatment on the Worsening of Periodontal Disease and Dental Caries: A Preliminary, Retrospective Study

**DOI:** 10.3290/j.ohpd.b1757253

**Published:** 2021-07-15

**Authors:** Sakiko Soutome, Mitsunobu Otsuru, Yumiko Kawashita, Madoka Funahara, Takashi Ukai, Toshiyuki Saito

**Affiliations:** a Associate Professor, Department of Oral Health, Nagasaki University Graduate School of Biomedical Sciences, Nagasaki, Japan. Idea, hypothesis, study design, wrote the manuscript, contributed substantially to the discussion.; b Senior Assistant Professor, Department of Clinical Oral Oncology, Nagasaki University Graduate School of Biomedical Sciences, Nagasaki, Japan. Idea, study design, contributed substantially to the discussion.; c Assistant Professor, Department of Oral Health, Nagasaki University Graduate School of Biomedical Sciences, Nagasaki, Japan. Idea, performed the study in partial fulfilment of requirements for a degree, contributed substantially to the discussion.; d Senior Lecturer, School of Oral Health Sciences, Kyushu Dental University, Fukuoka, Japan. Idea, study design, consulted on and performed statistical evaluation, contributed substantially to the discussion.; e Professor, Oral Management Centre, Nagasaki University Hospital, Nagasaki, Japan. Idea, study design, proofread the manuscript, contributed substantially to the discussion.; f Professor, Department of Oral Health, Nagasaki University Graduate School of Biomedical Sciences, Nagasaki, Japan. Idea, proofread the manuscript, contributed substantially to the discussion.

**Keywords:** chemotherapy, periodontal disease, perioperative oral management, radiotherapy, risk factor

## Abstract

**Purpose:**

To investigate the effect of cancer treatment on the worsening of dental caries and periodontal disease.

**Materials and Methods:**

Fifty-three adult cancer patients who underwent panoramic radiography before cancer treatment and 1–2 years later were enrolled in this study. They received professional oral care, including oral hygiene instruction, scaling and root planing, professional mechanical tooth cleaning, and dental treatment or extraction of any tooth with the source of infection. Age, sex, smoking habit, probing pocket depth, alveolar bone loss, oral hygiene, number of teeth, leukocytes, haemoglobin, and albumin counts, cancer treatment, the administration of immunosuppressants, worsening of dental caries, and alveolar bone loss after 1–2 years were examined. Factors related to the worsening of dental caries and alveolar bone loss were analysed using logistic regression analysis.

**Results:**

Dental caries and periodontal disease worsened in 20.8% of the patients. Smoking habit and chemotherapy were independent risk factors for the worsening of dental caries, while alveolar bone loss greater than 1/3 and chemotherapy were independent risk factors related to worsening periodontal disease.

**Conclusion:**

Anticancer drug treatment is an exacerbating factor for dental caries and periodontal disease.

Various oral complications associated with cancer therapy occur during and after surgery, radiotherapy, or chemotherapy.^13^ The most important cause of these complications is direct damage to the oral cavity (soft and hard tissues), weakened immune and other biological defence systems, or impaired healing mechanisms. They may also be caused by factors that have secondary effects, such as myelosuppression, weakened local immunity, and decreased saliva.^11^

Since 2012, perioperative oral management (POM) in Japan has been covered by medical insurance to prevent adverse events during cancer treatment, such as postoperative infections and stomatitis, by removing the source of oral infection and establishing good oral hygiene. When high-intensity chemotherapy that reduces the leukocyte count needs to be performed, teeth with apical lesions or severe periodontitis are often extracted before cancer treatment. However, teeth that were initially thought to be restorable may have be extracted due to the rapid deterioration of the periodontal condition or dental caries after cancer treatment.

Radiation therapy for head and neck cancer reportedly causes multiple dental caries because of its direct influence on teeth and secondary action due to decreased salivation.^1,9,10^ However, little is known about the effects of cancer treatment on periodontal disease, although cancer surgery, radiation therapy, and chemotherapy can reduce general and local immunity and may have adverse effects on the periodontal tissue.^8,11^ The length of time for which teeth can be preserved may differ between patients after cancer treatment and healthy individuals. Therefore, in order to perform POM, it is necessary to know whether dental diseases worsen faster during cancer treatment than in healthy people, and to identify the factors associated with the rapid deterioration of dental diseases. The purpose of this retrospective, preliminary study was to compare the initial oral findings with those 1-2 years later in cancer patients who underwent POM, determine the rate of deterioration of dental caries and periodontal disease, and investigate the risk factors associated with the exacerbations.

## Materials and Methods

### Patients

This retrospective observational study preliminarily examined the relationship between cancer treatment and worsening periodontal disease.

Two hundred fifty-nine (259) patients with an over 20-year-old malignant tumour visited the Oral Management Center of Nagasaki University Hospital for POM before cancer therapy (surgery, radiotherapy, or chemotherapy) between January and September 2018. Of these, 53 patients underwent POM at Nagasaki University Hospital for over a year and underwent panoramic radiography both before oral management and 1–2 years later. Other patients also underwent POM at Nagasaki University Hospital during hospitalisation, but were referred to other dentists after discharge. Patients under the age of 20 years and those without teeth were excluded from the study.

### Oral Care Intervention

Panoramic radiographs were taken for all patients and oral examinations for sources of infection such as dental caries, periodontal disease, and apical lesions were performed. In addition to providing oral hygiene guidance, scaling and root planing, and professional mechanical tooth cleaning, if teeth with the source of infection were present, dental treatment including tooth extraction was performed, taking into consideration the strength of cancer treatment or the period until the start of cancer treatment. In the case of radiation therapy for head and neck cancer, spacers to prevent radiation backscatter, administration of pilocarpine hydrochloride for xerostomia, steroid ointment for oral mucositis, and topical fluoride administration to prevent radiation-induced dental caries were also added.

### Variables

The following variables were examined using medical records and radiographic images: age, sex, smoking habit, probing pocket depth, alveolar bone loss (< 1/3 / ≥ 1/3) by panoramic radiography, oral hygiene (good/poor), number of teeth, minimum leukocyte count, haemoglobin level, and albumin count between the first and second panoramic radiographic examinations, surgery (+/–), chemotherapy (+/–), radiotherapy (+/–), and the administration of immunosuppressants such as corticosteroids or anti-rheumatic drugs (+/–). A plaque control record < 20% was classified as good and ≥20% as poor. Probing pocket depth and alveolar bone loss were evaluated in the most severely affected teeth. When tooth extraction was performed before cancer treatment, the number of teeth were counted after tooth extraction.

### Endpoints

The primary endpoint was worsening of alveolar bone loss as seen in panoramic radiographs. The secondary endpoint was worsening of dental caries. When new dental caries occurred or clinical examination revealed that caries progressed, it was defined as worsening of caries. Panoramic radiographs of the patients were taken before and 1–2 years after cancer treatment (mean ± SD; 562 ± 156 days).

### Statistical Analysis

The relationship between the worsening of alveolar bone loss or dental caries and each variable was analysed using Fisher’s exact test or one-way ANOVA, followed by multivariate analysis using logistic regression analysis. All statistical analyses were performed using SPSS version 26.0 (Japan IBM; Tokyo, Japan). A p-value < 0.05 was considered statistically significant.

### Ethics and Registration

The study was approved by the institutional review board of Nagasaki University Hospital, and the research plan and guaranteed opt-out opportunity were published on the homepage of the official website of the hospital. As this was a retrospective observational study, it was not registered.

### Data Availability

The datasets used and analysed during the study are available from the corresponding author upon reasonable request.

## Results

### Patient Characteristics

Forty-two patients were male and 11 were female, with an average age of 65.5 years. The primary disease was head and neck cancer in 22 patients, gastrointestinal cancer in 15, lung cancer in 6, renal urinary cancer in 3, breast cancer in 2, and other cancer in 5 patients. Forty-three patients underwent surgery, 20 underwent radiotherapy, and 25 underwent chemotherapy. All 20 patients who underwent radiotherapy had head and neck cancer (Table 1).

**Table 1:** Patient characteristics

Factors	Category	Number of patients / mean ± SD
Age		65.5 ± 12.0
Sex	male	42
	female	11
Primary disease	head and neck cancer	22
	gastrointestinal cancer	15
	lung cancer	6
	renal urinary cancer	3
	breast cancer	2
	other cancer	5
Smoking habit	(–)	23
	(+)	30 (BI*: 874 ± 758)
Probing pocket depth > 4 mm	(–)	25
	(+)	28
Maximum probing pocket depth		4.21 ± 1.68
Alveolar bone loss	< 1/3	46
	≥ 1/3	7
Oral hygiene	good	21
	poor	32
Number of teeth		20.3 ± 8.01
Leukocytes		3632 ± 1459
Hemoglobin		10.4 ± 2.00
Albumin		2.91 ± 0.688
Surgery	(–)	10
	(+)	43
Radiotherapy	(–)	20
	(+)	33
Chemotherapy	(–)	28
	(+)	25
Immunosuppressants	(–)	48
	(+)	5

*BI: Brinkman Index

### Factors Related to Worsening Dental Caries after Cancer Treatment

Dental caries worsened in 11 patients (20.8%) one to two years after cancer therapy. The degree of dental caries changed from no dental caries to C2 (dental caries in dentin but not pulp) in 4 cases, from C1 (dental caries limited to enamel) to C2 in 5 cases, from C2 to C4 (only a stump/residual root left) in one case, and from C3 (dental caries in the pulp) to C4 in one case. The rate of deterioration in those undergoing radiotherapy for head and neck cancer was 5/20 (25.0%), which was higher than 6/33 (18.2%) in those who did not undergo radiotherapy, but the difference was not statistically significant. Univariate analysis revealed that patients with a smoking habit (p = 0.015) and those who received anticancer drugs (p = 0.050) had a statistically significantly higher rate of dental caries exacerbation, although other variables did not correlate with worsening dental caries (Table 2).

**Table 2:** Relationship between each variable and caries (univariate analysis)

Variable		Change in caries		
		No change	Progression	p-value
Age	years	64.6 ± 12.2	68.9 ± 11.5	0.297
Sex	male	15	2	0.469
	female	27	9	
Smoking habit	(–)	22	1	*0.015
	(+)	20 (BIǂ: 919 ± 882)	10 (BIǂ: 785 ± 445)	
Periodontal pocket > 4 mm	(–)	19	6	0.737
	(+)	23	5	
Maximum probing pocket depth		4.26 ± 1.74	4.00 ± 1.48	0.650
Alveolar bone loss	<1/3	38	8	0.147
	≥1/3	4	3	
Oral hygiene	good	18	3	0.494
	poor	24	8	
Number of teeth		22.2 ± 7.35	20.2 ± 7.14	0.426
Leukocytes	/μL	3579 ± 1461	3836 ± 1504	0.607
Hemoglobin	g/dL	10.4 ± 2.03	10.2 ± 1.96	0.820
Albumin	g/dL	2.91 ± 0.728	2.93 ± 0.546	0.941
Surgery	(–)	7	3	0.416
	(+)	35	8	
Radiotherapy	(–)	27	6	0.728
	(+)	15	5	
Chemotherapy	(–)	26	3	*0.050
	(+)	16	8	
Immunosuppressants	(–)	37	11	0.571
	(+)	5	0	
Total		42	11	

ǂBI: Brinkman Index. *Statistically significant.

Multivariate analysis also showed that smoking habit (p = 0.018, odds ratio = 14.832) and chemotherapy (p = 0.027, odds ratio = 6.113) were independent risk factors for the worsening of dental caries (Table 3).

**Table 3:** Relationship between each variable and progression of caries (multivariate analysis)

Variable		p-value	Odds ratio	95% confidence interval
Smoking habit	(–) vs (+)	*0.018	14.832	1.596–137.808
Chemotherapy	(–) vs (+)	*0.027	6.113	1.230–30.381

*Statistically significant.

### Factors Related to Worsening Periodontal Disease after Cancer Treatment

Periodontal disease worsened in 11 patients (20.8%), and the rate of deterioration was statistically significantly higher in patients showing alveolar bone loss >1/3 (p < 0.001) and in those undergoing chemotherapy (p = 0.015) (Table 4).

**Table 4:** Relationship between each variable and periodontal disease (univariate analysis)

Variable		Change in periodontal disease		
		No change	Progression	p-value
Age	years	64.1 ± 12.1	70.9 ± 10.6	0.095
Sex	male	14	3	1.000
	female	28	8	
Smoking habit	(–)	20	4	0.484
	(+)	23 (BI: 912 ± 835)	7 (BI: 750 ± 448)	
Periodontal pocket > 4 mm	(–)	20	5	1.000
	(+)	22	6	
Maximum probing pocket depth		4.10 ± 1.56	4.64 ± 2.11	0.346
Alveolar bone loss	< 1/3	41	5	*<0.001
	≥ 1/3	1	6	
Oral hygiene	good	17	4	1.000
	poor	25	7	
Number of teeth		21.9 ± 7.39	21.2 ± 7.15	0.773
Leukocytes	/μL	3743 ± 1475	3209 ± 1378	0.284
Hemoglobin	g/dL	10.6 ± 1.99	9.42 ± 1.83	0.076
Albumin	g/dL	2.98 ± 0.729	2.68 ± 0.464	0.212
Surgery	(–)	6	4	0.187
	(+)	36	7	
Radiotherapy	(–)	28	6	0.496
	(+)	14	5	
Chemotherapy	(–)	27	2	*0.015
	(+)	15	9	
Immunosuppressants	(–)	38	10	1.000
	(+)	4	1	
Total		42	11	

BI: Brinkman Index. *Statistically significant.

Multiple regression analysis revealed that alveolar bone loss > 1/3 (p = 0.005, odds ratio = 66.155) and chemotherapy (p = 0.043, odds ratio = 10.571) were independent risk factors for the worsening of periodontal disease (Table 5).

**Table 5:** Relationship between each variable and progression of periodontal disease (multivariate analysis)

Variable		p-value	Odds ratio	95% confidence interval
Alveolar bone loss	< 1/3 vs ≥ 1/3	*0.005	66.155	3.587–1220.242
Chemotherapy	(–) vs (+)	*0.043	10.571	1.081–103.415

*Statistically significant.

## Discussion

Various oral complications may occur during and after cancer therapy. Early oral complications include oral mucositis caused by high-intensity anticancer drugs and radiation therapy to the head and neck.^3,6^ Chemotherapy and radiation therapy can reduce systemic and local immunity, which may lead to acute exacerbations of chronic dental infections and sometimes sepsis. Hong et al^2^ conducted a systematic review of dental disease management in cancer patients, and stated that the weighted prevalence of dental infections and pericoronitis during cancer therapy was 5.4% and 5.3%, respectively. In a multicentre retrospective study, we also reported that acute dental focal infection, such as acute periodontitis and alveolar abscess, occurred during the period of cancer treatment in 8.2% of 2744 cancer patients, and in 113 patients (4.1%), prolonged fever was observed, with 7 having dental focal infections. Therefore, we concluded that POM was important for the safe completion of cancer treatment.^13^ Radiation therapy for head and neck cancer also causes various oral complications. Oral soft tissue changes caused by radiation therapy include oral mucositis during and soon after treatment, mucosal opportunistic infections, neurosensory disorders, and tissue fibrosis.^10^ Late severe adverse effects on the hard tissue include radiation-induced dental caries^8^ and osteoradionecrosis of the jaw (ORN).^4^ No statistically significant association between radiation therapy and dental caries was found in this study, probably because patients undergoing radiation therapy received topical fluoride in addition to regular dental interventions.

The effect of cancer treatment on periodontal disease, however, is not well understood. Vargas-Villafuerte et al^12^ reported that patients with breast cancer who were undergoing chemotherapy responded to periodontal non-surgical therapy, although with less favourable results than those with periodontitis without cancer, and may require additional or adjunctive periodontal treatment. Quispe et al^8^ also stated that periodontal disease, edentulism, and a greater need for oral rehabilitation were the most significant changes in individuals who received antineoplastic therapy for head and neck cancer. Conversely, Muthular et al^5^ reported that tamoxifen, an anticancer drug for breast cancer, chronically improved periodontal health and had antifungal activity against oral strains isolated from patients with odontogenic and medical pathologies.

This study revealed that anticancer drug treatment is one of the exacerbating factors for periodontal disease. Oral hygiene level and periodontal status at baseline were not associated with worsening periodontal disease 1–2 years after cancer treatment. This is thought to be because factors which reduce systemic and local immunity, e.g. chemotherapy, have a greater effect on the worsening of periodontal disease than factors such as oral hygiene, which are usually the cause of periodontal disease. Chemotherapy may reduce the amount and type of pathogenic microorganisms in the periodontal pocket, resulting in worsening periodontal disease.^7^ Furthermore, toothbrushing may be neglected due to nausea caused by the side effects of anticancer drugs, although no such report exists in the literature. We are preparing a prospective study on the bacterial flora and cytokine production in the probing pocket depth in patients with periodontal disease who are being treated with anticancer drugs. If the risk factors for the exacerbation of periodontal disease in patients undergoing cancer treatment are understood, it may lead to the establishment of appropriate oral management methods for patients with these factors.

This study had several limitations. First, it was a preliminary study based on a small number of cases in a single facility, thus making it difficult to generalise the results obtained. Furthermore, because it was a retrospective observational study, oral management interventions could not be unified and the effects of treatment could not be ruled out.

## Conclusion

This study established that anticancer drug treatment is an exacerbating factor for dental caries and periodontal disease. Periodontal disease worsened more rapidly in patients who received anticancer drugs than in those who did not. In the future, we plan to investigate the effects of anticancer drugs on periodontal tissues in detail by a multicenter, prospective study.

## References

[ref1] Gupta N, Pal M, Rawat S, Grewal MS, Garg H, Chauhan D (2015). Radiation-induced dental caries, prevention and treatment – A systematic review. Natl J Maxillofac Surg.

[ref2] Hong CHL, Hu S, Haverman T, Stokman M, Napeñas JJ, Braber JB (2018). A systematic review of dental disease management in cancer patients. Support Care Cancer.

[ref3] Kawashita Y, Koyama Y, Kurita H, Otsuru M, Ota Y, Okura M (2019). Effectiveness of a comprehensive oral management protocol for the prevention of severe oral mucositis in patients receiving radiotherapy with or without chemotherapy for oral cancer: a multicentre, phase II, randomized controlled trial. Int J Oral Maxillofac Surg.

[ref4] Kojima Y, Yanamoto S, Umeda M, Kawashita Y, Saito I, Hasegawa T (2017). Relationship between dental status and development of osteoradionecrosis of the jaw: a multicenter retrospective study. Oral Surg Oral Med Oral Pathol Oral Radiol.

[ref5] Muthular M, Bálsamo F, Passero P, Jewtuchowicz V, Miozza V, Villalba B (2019). Effects of tamoxifen on periodontal disease and Candida albicans of patients with breast cancer and other pathologies. Future Microbiol.

[ref6] Nishii M, Soutome S, Kawakita A, Yutori H, Iwata E, Akashi M (2020). Factors associated with severe oral mucositis and candidiasis in patients undergoing radiotherapy for oral and oropharyngeal carcinomas: a retrospective multicenter study of 326 patients. Support Care Cancer.

[ref7] Peterson DE, Minah GE, Overholser CD, Suzuki JB, DePaola LG, Stansbury DM (1987). Microbiology of acute periodontal infection in myelosuppressed cancer patients. J Clin Oncol.

[ref8] Quispe RA, Cremonesi AL, Gonçalves JK, Rubira CMF, Santos PSDS (2018). Case-control study of oral disease indexes in individuals with head and neck cancer after antineoplastic therapy. Einstein (Sao Paulo).

[ref9] Soutome S, Funahara M, Hayashida S, Nakamura K, Umeda M (2017). Risk factors for radiation-induced dental caries in patients with head and neck cancer. Oral Health Care.

[ref10] Springer IN, Niehoff P, Warnke PH, Böcek G, Kovács G, Suhr M (2005). Radiation caries-radiogenic destruction of dental collagen. Oral Oncol.

[ref11] Sroussi HY, Epstein JB, Bensadoun RJ, Saunders DP, Lalla RV, Migliorati CA (2017). Common oral complications of head and neck cancer radiation therapy: mucositis, infections, saliva change, fibrosis, sensory dysfunctions, dental caries, periodontal disease, and osteoradionecrosis. Cancer Med.

[ref12] Vargas-Villafuerte KR, Dantas FT, Messora MR, Novaes Jr AB, Grisi MF, Taba Jr M (2016). Preliminary results of non-surgical periodontal treatment in patients with breast cancer undergoing chemotherapy. J Periodontol.

[ref13] Yamada S, Soutome S, Hasegawa T, Tojyo I, Nakahara H, Kawakami M (2020). A multicenter retrospective investigation on the efficacy of perioperative oral management in cancer patients. Medicine (Baltimore).

